# Alpha-fetoprotein-producing ovarian clear cell adenocarcinoma with fetal gut differentiation: a rare case report and literature review

**DOI:** 10.1186/s13048-018-0426-8

**Published:** 2018-06-22

**Authors:** Wei-Ting Chao, Chia-Hao Liu, Chiung-Ru Lai, Yi-Jen Chen, Chi-Mu Chuang, Peng-Hui Wang

**Affiliations:** 1 Faculty of Medicine, College of Medicine, Fu-Jen Catholic University, Taipei, Taiwan; 2 Department of Obstetrics and Gynecology, Taipei Veterans General Hospital, No. 201, Section 2, Shih-Pai Road, Taipei, 112 Taiwan; 3 Department of Obstetrics and Gynecology, National Yang-Ming University School of Medicine, Taipei, Taiwan; 4 Department of Pathology, Taipei Veterans General Hospital, Taipei, Taiwan; 5 Department of Midwifery and Women Health Care, National Taipei University of Nursing and Health Sciences, Taipei, Taiwan; 6 Department of Medical Research, China Medical University Hospital, Taichung, Taiwan

**Keywords:** Alpha-fetoprotein, Ovarian clear cell adenocarcinoma, Fetal gut differentiation

## Abstract

**Background:**

Alpha-fetoprotein (AFP) is a useful tumor marker for ovarian germ cell tumors, particularly yolk sac tumor (YST). It is valuable for both diagnosis and further follow-up. Epithelial ovarian carcinoma (EOC) rarely secretes AFP, especially for clear cell type and in the postmenopausal women. Based on the limited knowledge about AFP-producing clear cell type EOC, a case and literature review on this topic is extensively reviewed.

**Case presentation:**

We report a 55-year-old postmenopausal woman experienced vaginal spotting for one month, and serum level of AFP was 60,721 ng/ml initially. Histological examination was clear cell type EOC. Tumor cells revealed strong immunoreactivity for glypican-3 (GPC3) and AFP and weak for hepatocyte nuclear factor-1 beta (HNF-1 beta), but negative for CD30, making the diagnosis of AFP-producing clear cell type EOC with fetal gut differentiation in focal areas, FIGO (International Federation of Gynecology and Obstetrics) IIIc. Although the patient underwent an intensive treatment, including optimal debulking surgery and multi-agent chemotherapy, the patient died of disease. To provide a better understanding of clinical and molecular characteristics of the AFP-producing clear cell type EOC, we conducted a systematic literature review.

**Conclusions:**

A total of three papers described the AFP-producing clear cell type EOC are available. The overall survival rate of these cases, including the current case is 50%. Although immunohistochemical examination is not always needed in routine for the diagnosis of clear cell type EOC, to distinguish from other tumors, especially germ cell tumors, or to provide the better way to monitor therapeutic response or to evaluate the disease status, immunostaining, including GPC3, HNF-1 beta, CD30, cytokeratin 7 or 20, and AFP is taken into account. Due to rarity, the appropriate chemotherapy regimen and the biological behavior of AFP-producing clear cell type EOC are still unclear.

## Background

Epithelial ovarian carcinoma (EOC) rarely secretes alpha-fetoprotein (AFP) in postmenopausal women, especially for clear cell type. We report a 55-year-old postmenopausal woman with AFP-producing clear cell type EOC with fetal gut differentiation, FIGO (International Federation of Gynecology and Obstetrics) IIIC, and conducted a systematic literature review for published cases of AFP-producing clear cell type EOC. To our knowledge, this is the first literature review of AFP-producing clear cell type EOC.

## Case presentation

**Fig. 1:**
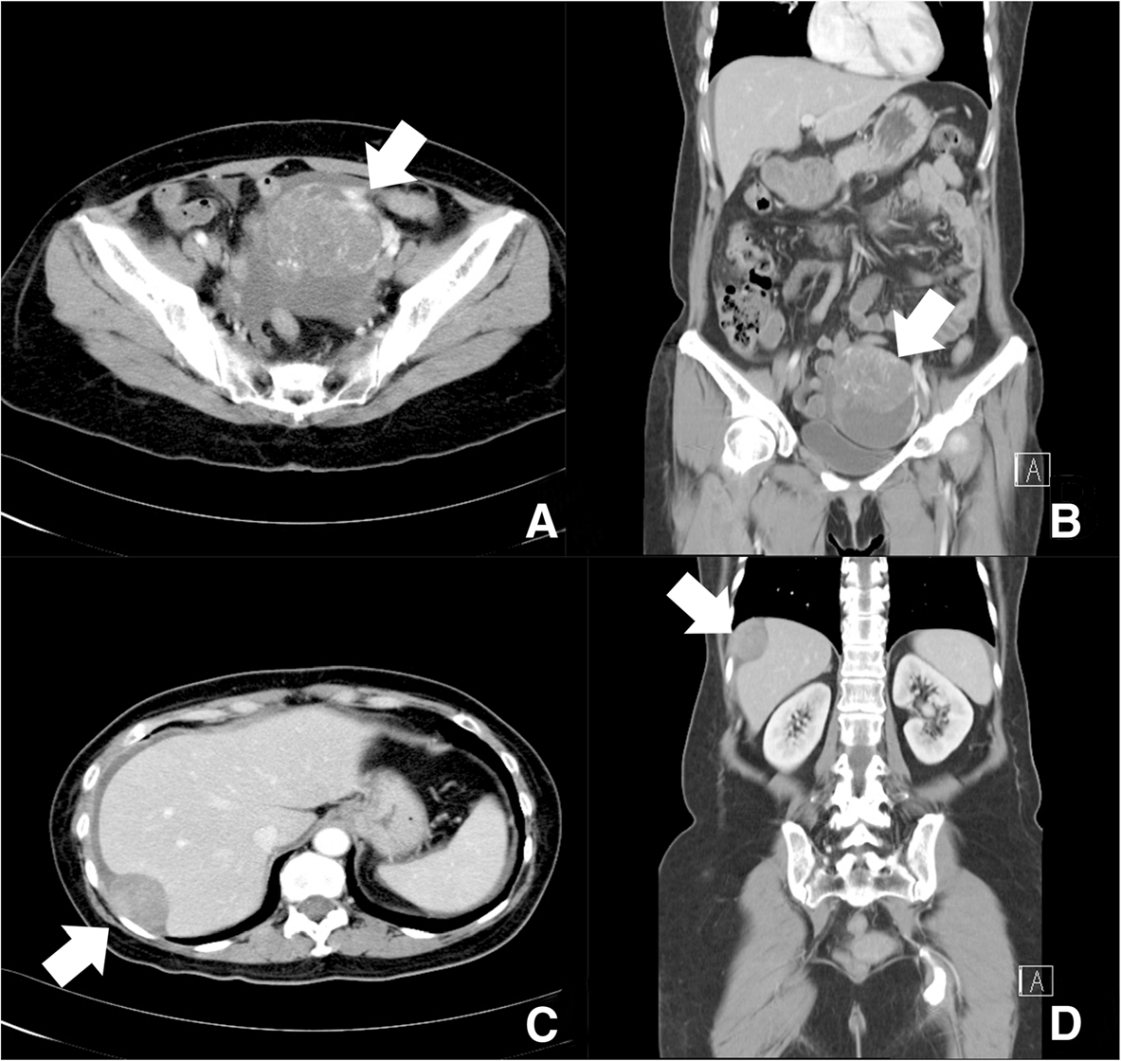
Computed tomography (**a**) axial and (**b**) coronal view showing a heterogenous mass lesion measuring 9 cm in diameter that probably developed from the left adnexa. **c** axial and (**d**) coronal view showing a well-defined mass measuring 4 cm in diameter located at the right subphrenic region

A 52-year-old menopausal woman complained of intermittent vaginal spotting for 1 month. She denied any systemic disease, dysmenorrhea, menorrhagia, body weight loss, abdominal pain, or abdominal fullness. Gynecologic history was gravida 2 and para 2. Transvaginal ultrasound revealed a 10-cm multi-lobular cystic pelvic mass containing the mixed heterogeneous solid component, fluid and papillary growth in the inner surface of cystic wall Significant venous flow was detected in the solid part and papillary growth lesion. Serum AFP (< 20 ng/ml), cancer antigen (CA)-125 (< 35 U/ml), and carcinoembryonic antigen (< 5 ng/ml), and CA19–9 (< 37 U/ml) were 60,721, 38.1, 84, and 97 ng/ml, respectively. All of these tumor markers from serum have their own specific cut off values and sensitivities, and they come from the same assay methods and from the same laboratory. All are elevated. Computed tomography (CT) showed a 9-cm heterogenous mass probably developed from the left adnexa (Fig. 1a, b) and a 4-cm well-defined mass located at the right subphrenic region (Fig. 1c, d), suggesting the diagnosis of left ovarian carcinoma with peritoneal seeding.

**Fig. 2:**
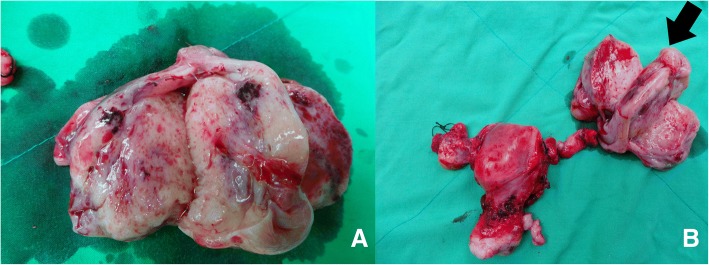
**a** Left ovarian solid tumor with cystic component measuring 9 × 5 × 5 cm attached to the left fallopian tube. **b** Solid tumor in the uterus measuring 8 × 5 × 3.5 cm attached to the right ovary: 2.5 × 2 × 1 cm, right fallopian tube (5 cm in length), left ovarian tumor, and left fallopian tube

**Fig. 3:**
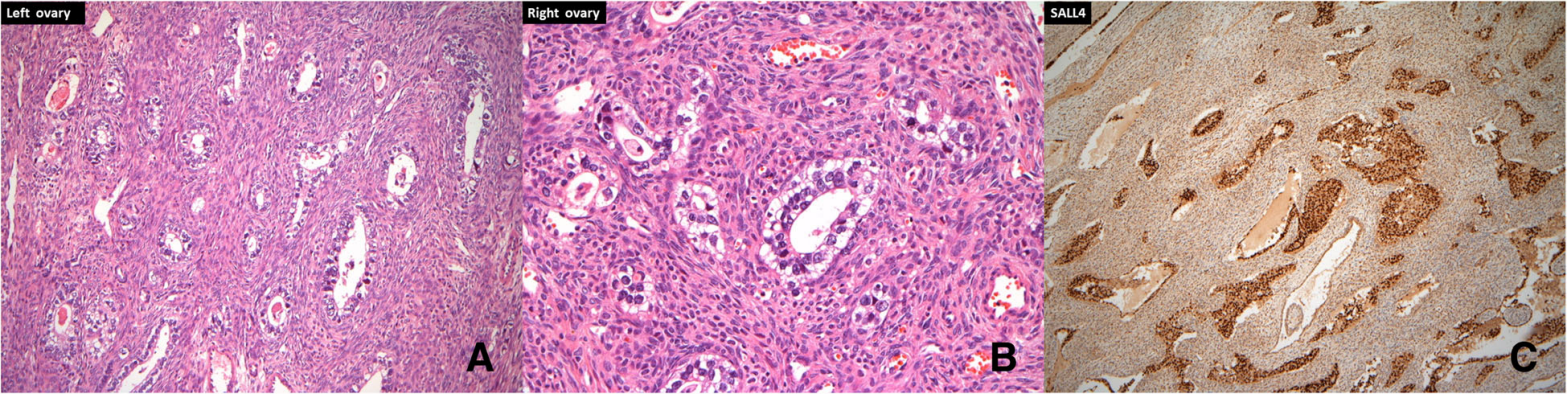
**a** Hematoxylin–eosin stain. Microscopically, the left ovarian tumor was a clear cell carcinoma, × 200. **b** Hematoxylin–eosin stain. Microscopically, the right ovarian tumor was metastatic clear cell carcinoma, × 200. **c** SALL4, which are known as oncofetal proteins expressed in germ cell tumors, showed positive staining, × 400

**Fig. 4:**
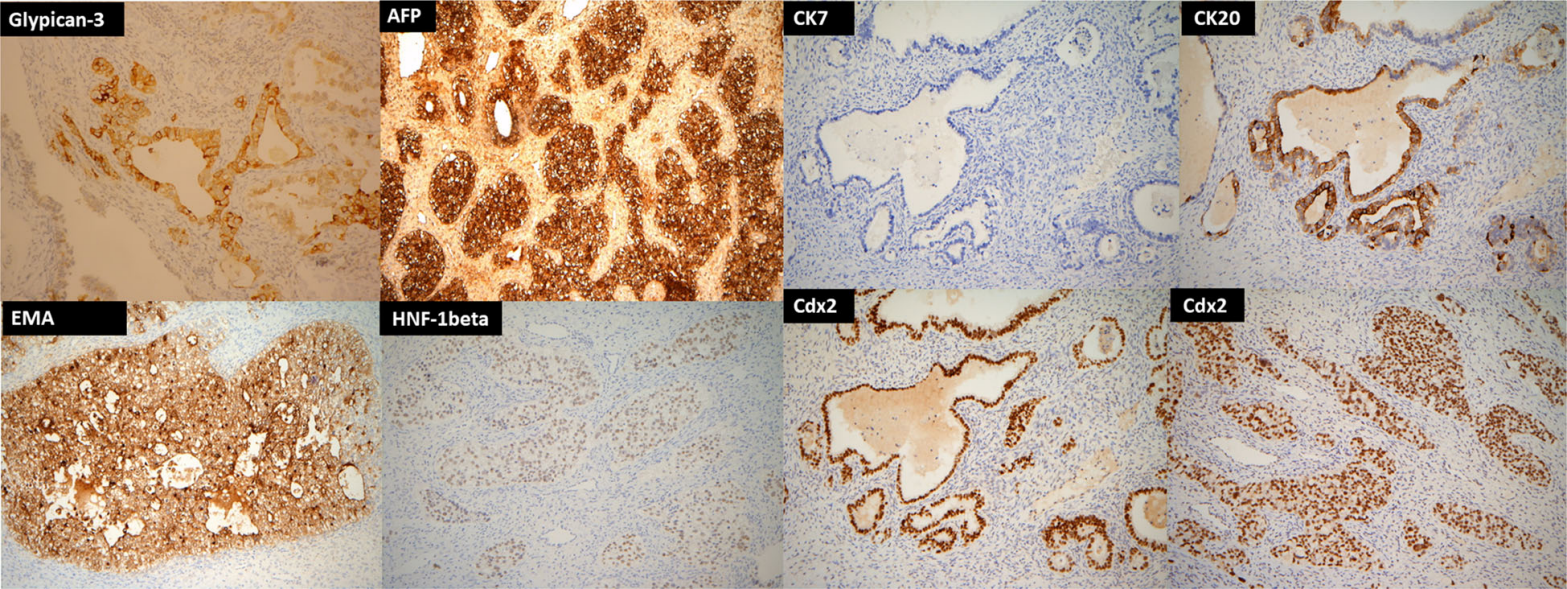
Tumor cells were positive for GPC3, AFP, and EMA. The tumor cells showed weak positive staining for HNF-1beta, while focally positive for cytokeratin (CK)20 and Cdx2 and negative for CK7, × 400

**Fig. 5:**
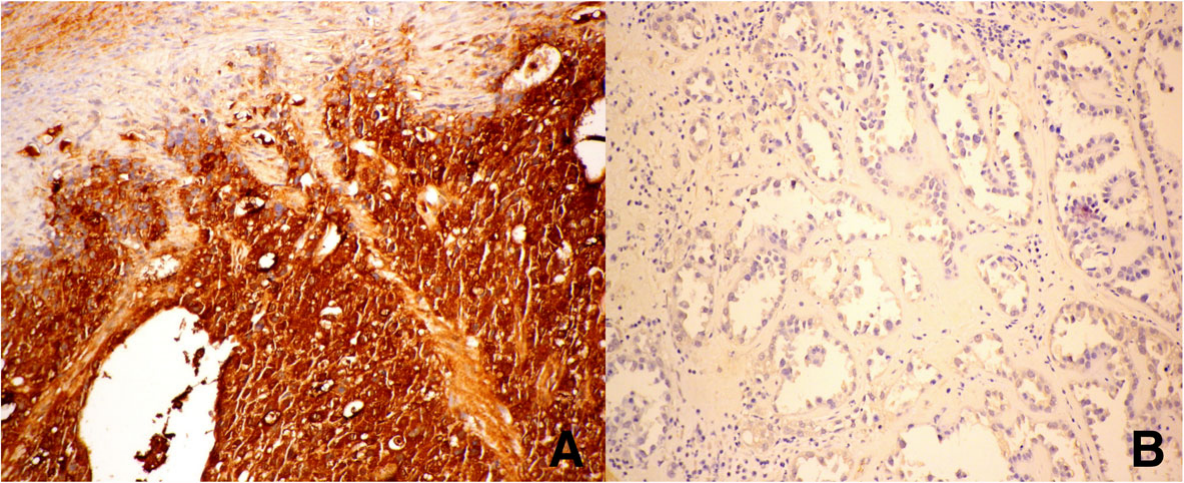
**a** We titrated the antibody of AFP staining. Tumor cells were strongly positive for AFP staining while it was negative in background stroma, × 100. **b** Negative control in classical clear cell carcinoma, × 100

The patient underwent optimal debulking surgery, including total hysterectomy, bilateral salpingo-oophorectomy, omentectomy, pelvic lymphadenectomy, and para-aortic lymphadenectomy (Fig. 2a, b). All gross tumors were almost completely resected. Histologically, sections of the left ovarian tumor showed a clear cell carcinoma (Fig. 3a). The tumor is composed of polygonal, cuboidal to columnar cells with clear cytoplasm arranged in solid nests and tubule-cystic growth patterns. Numerous hyaline globules are present. Some tumor cells also showed high-grade anaplastic nuclear features. Right ovarian tumor showed metastatic clear cell carcinoma (Fig. 3b). Typical yolk sac tumor differentiation and Shiller-Duval body were absent. Sal-like protein 4 (SALL4) was strongly positive (Fig. 3c). Immunohistochemically, the tumor cells were positive for glypican-3 (GPC3) and AFP and negative for CD30. Meanwhile, the tumor cells showed weak positive staining for hepatocyte nuclear factor1-beta (HNF-1beta) (Fig. 4). The tumor cells were focally positive for cytokeratin (CK) 20 and Cdx2 (caudal type homeobox 2) and negative for CK7 (Fig. 4). To rule out potential artifacts due to antibody issues, we titrated the antibody used for AFP staining (Fig. 5a). For every immunohistochemical stain, positive controls were included. As a negative control, we also stained the classical clear cell carcinoma (Fig. 5b). Combination of all made a diagnosis of AFP-producing clear cell type EOC with fetal gut differentiation, FIGO (International Federation of Gynecology and Obstetrics) IIIc.

**Fig. 6:**
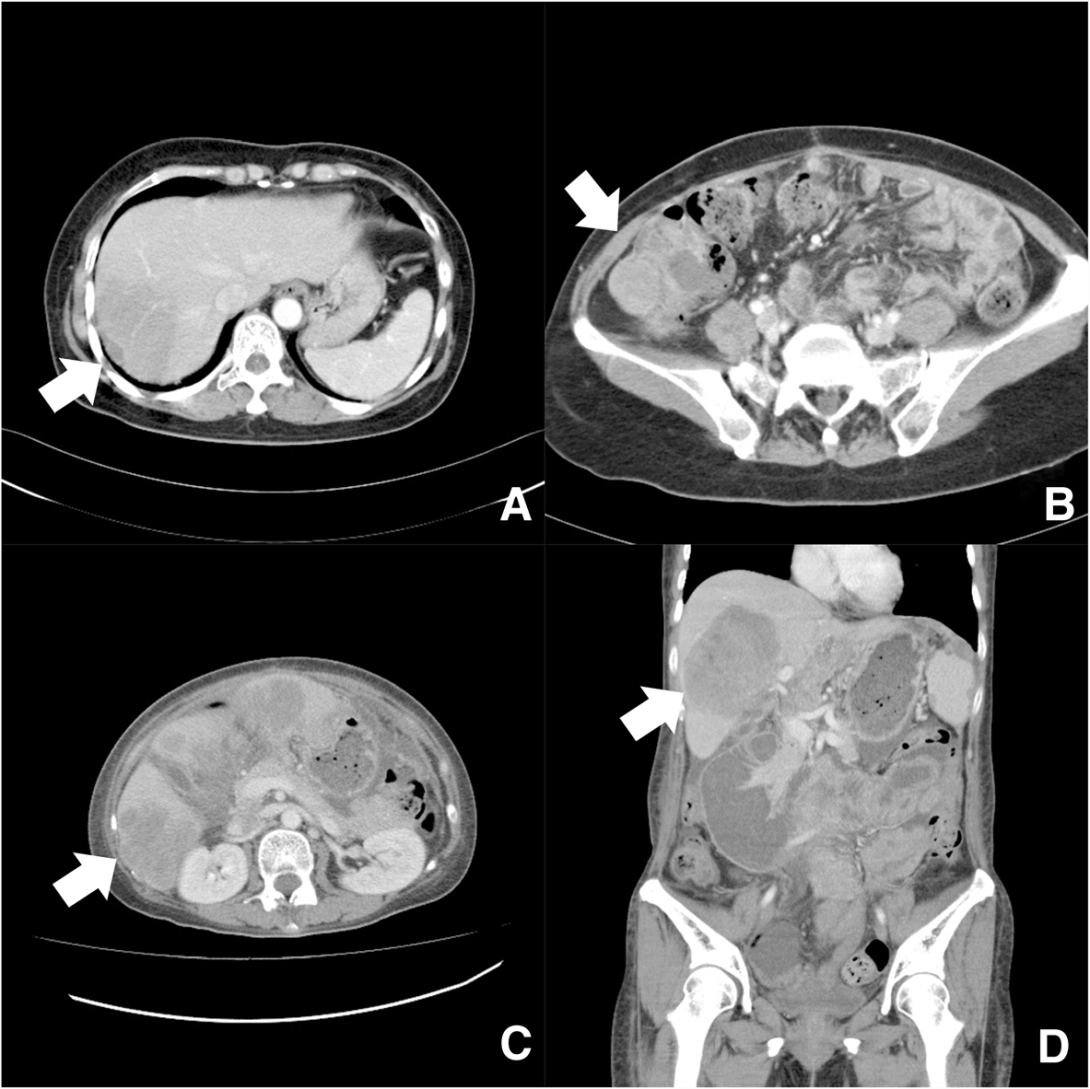
Computed tomography (**a**) axial view showing a 2-cm hypodense nodule at the right subphrenic region and wedge-shaped hypoperfusion over segment 7 of the liver suspicious tumor seeding and liver metastasis. **b** Coronal view showing a lobulated mixed solid and cystic lesion at the right paracolic gutter measuring 5 cm in size in favor of peritoneal seeding. **c** Axial and (**d**) coronal view on further abdominal CT follow-up showing tumor recurrence of the liver and increased tumor burden after 18 months of tertiary debulking surgery

After surgery, the patient received adjuvant multi-agent chemotherapy (carboplatin and paclitaxel). After treatment, CT showed a 2-cm hypodense nodule at the right subphrenic region and wedge-shaped hypoperfusion over segment 7 of the liver, suspicious tumor seeding, and liver metastasis (Fig. 6a) and serum level of AFP was 1831 ng/ml. Tumor excision for liver metastasis was done. Dose-intensity chemotherapy with weekly paclitaxel was given. CT scan, 1 year later, showed a lobulated mixed solid and cystic lesion at the right paracolic gutter measuring 5 cm in size in favor of peritoneal seeding (Fig. 6b). Exploratory laparotomy was done, including the use of Cavitron Ultrasonic Surgical Aspirator. After operation, chemotherapy was given through intraperitoneal route. After additional 18 months, tumor recurrence was found, including persistent liver metastases (Fig. 6c, d), with increasing serum level of AFP of 330,014 ng/ml. The patient finally died of disease.

### Literature search strategy

Based on our search of PubMed (from January 1960 to July 2018; search terms: “Alpha-fetoprotein”, “clear cell”, “ovarian cancer”; https://www.ncbi.nlm.nih.gov/pubmed/?term=Alpha-fetoprotein%2C+clear+cell%2C+ovarian+cancer), there are a few cases of AFP-producing clear cell type EOC available in the literature [1–3].

### Results

A total of three articles are included and the detailed information, including microscopic finding and immunohistochemical staining results are summarized in Table 1.

**Table 1:** Summary of Alpha-fetoprotein producing clear cell type epithelial ovarian cancer

Author	Year of publication	Age	Size	FIGO stage	Microscopic findings and immunohistochemical stain	Outcome
Cetin et al. [1]	2006	63	22 cm	IIIc	There were polyhedral cells with clear cytoplasm and a pleomorphic nucleus. AFP(+), EMA(+), Pancytokeratin(+) CA-125(−), CK 20(−),Vimentin(−)	Dead 70 months
Takahashi et al. [2]	2010	54	20 cm	IIIc	There were papillary, tubular, and cribriform growths of atypical glandular cells having pale cytoplasm, distinct cellular border and enlarged atypical nuclei with prominent nucleoli. Typical yolk sac tumor differentiation and Shiller–Duval body were absent. AFP(+)	Disease free
Morimoto et al. [3]	2014	59	14 cm	IIb	The presence of cuboidal or polygonal cells having a clear cytoplasmin solid nests and intermediate nuclear grade. No typical hobnail cells of conventional CCA or Schiller Duval bodies commonly observed in YST were detected. AFP(+), AE1/3(+), CEA(+), LeuM1(+), GPC3(+), Ki-67(+), SALL4(+), Calretinin(−), CD30(−), CK7(−), C-kit(−), EMA(−), HCG(−), HNF-1β(−), Inhibin-α(−), PLAP(−)	Disease free
Present case	2018	55	9 cm	IIIc	Polygonal, cuboidal to columnar cells with clear cytoplasm arranged in solid nests and tubule-cystic growth patterns AFP(+), CK20(+), Cdx2(+), GPC3(+), HNF-1beta(+), SALL4(+) CD30(−), CK7(−)	Dead 43 months

All patients were middle aged (range, 54–63 years). Tumor size ranged from 14 cm to 22 cm, but all were advanced FIGO stage (IIb–IIIc). Among these [1–3], including the current case, half of patients died of disease.

## Discussion

Production of AFP in tumors us often found in the germ cell tumors, particularly yolk sac tumor [4, 5]. AFP is also secreted in other tumors such as hepatocellular carcinoma and testicular carcinoma [6]. Epithelial ovarian cancers had been rarely reported to secrete AFP [7, 8], and AFP-producing EOCs are also extremely rare. Meguro et al. reported a comprehensive review addressing the topic of AFP-producing ovarian tumors with germ cell differentiation in postmenopausal women [9]. A total of 18 cases were included, and all patients were middle-aged (57–76 years). Histopathological findings were varied as follows: 9 hepatoid carcinoma [10–16], 4 endometrioid adenocarcinoma [8, 17–19], 3 mucinous adenocarcinoma [7, 20, 21], 2 serous adenocarcinoma [22, 23], 1 clear cell carcinoma [1], and 1 uncertain [24].

Histopathological examination in the current case showed tumors largely comprising clear cells and lacking the germ cell tumor component. The tumor was composed of polygonal, cuboidal to columnar cells with fetal gut differentiation. The CK7/CK20 pattern of the tumors is the most helpful immunochemical marker because the CK7-/CK20+ pattern is typical of the diagnosis of colorectal adenocarcinomas [25]. Cdx2, a critical nuclear transcription factor for intestinal development, is expressed in intestinal epithelium and adenocarcinomas [26]. The presence of Cdx2 in the current case report was indicative of fetal gut differentiation. These cells in the current case were positive for GPC3 and SALL4, which are known as oncofetal proteins expressed in germ cell tumors [3, 27], contributing to the need of additional immunohistochemical staining for differentiating germ cell tumors from clear cell type EOC in the current case. HNF-1 beta is a transcription activator that regulates genes expressed specifically in the liver-specific, such as albumin and AFP [28]. HNF-1 beta is reported to serve as clear cell type EOC marker with a high sensitivity [29–31], especially when the diffuse and strong HNF-1 beta expression pattern is found [30]. In the current case, the diagnosis of clear cell type EOC is based on this finding. Taken together, the combination of morphological and immunohistochemical findings confirmed the diagnosis of AFP-producing ovarian clear cell carcinoma with fetal gut differentiation.

AFP-producing clear cell type EOC has two characteristics: an ovarian clear cell carcinoma with and without typical germ cell tumor. AFP production in some EOCs is attributed to the foci of yolk sac tumors, such as endometrioid adenocarcinoma with a yolk sac component, mucinous adenocarcinoma with a yolk sac component, clear cell adenocarcinoma with a serous adenocarcinoma component, and endometrioid adenocarcinoma with a clear cell component [1, 8, 17, 19, 27].

However, the current case was largely composed of clear cells that lacked the germ cell tumor component. The elevation of serum tumor markers, such as CA-125 and AFP in the current case has been found in previous studies [1, 2]. Similar to Dr. Cetin’s report [1], AFP seemed to be more sensitive for tumor follow-up compared to CA-125. Meanwhile, Takahashi et al. presented a case in which CA-125 elevation was correlated with ovarian clear cell malignancy, and AFP elevation was due to hepatoid differentiation or neometaplasia from clear cell carcinoma [2]. By contrast, Morimoto et al. reported a case of ovarian clear cell adenocarcinoma with elevated AFP without elevated CA-125 [3].

Extensive surgery is the primary treatment modality due to diagnosis of EOC in the current case. Although we followed the current concept to use the combination of platinum and paclitaxel in the management of this patient, which has been shown by Takahashi et al. [2] and Morimoto et al. [3], the patient still died of disease. The possible reason might be related to advanced disease status (FIGO IIIC) [32], clear cell type [33], and AFP-producing component, and all of them might contribute to worse outcome of the current case.

## Conclusion

Histopathological and advanced immunohistochemical examination, such as for GPC3, SALL4, and HNF-1 beta are crucial for the current diagnosis of AFP-producing clear cell type EOC with gut differentiation. Due to rarity of similar cases, an optimal treatment, especially for the regimen of chemotherapy is still uncertain.

## Abbreviations

AFP: Alpha-fetoprotein
Cdx2: Caudal type homeobox 2
CEA: Carbohydrate antigen
CK: Cytokeratin
CT: Computed tomography
FIGO: International Federation of Gynecology and Obstetrics
GPC3: Glypican-3
HNF-1beta: Hepatocyte nuclear factor-1 beta
YST: Yolk sac tumor

## References

[1] Cetin A, Bahat Z, Cilesiz P, Demirbag N, Yavuz E (2007). Ovarian clear cell adenocarcinoma producing alpha-fetoprotein: case report. Eur J Gynaecol Oncol.

[2] Takahashi Y, Mogami H, Hamada S, Urasaki K, Konishi I (2011). Alpha-fetoprotein producing ovarian clear cell carcinoma with a neometaplasia to hepatoid carcinoma arising from endometriosis: a case report. J Obstet Gynaecol Res.

[3] Morimoto A, Sudo T, Sakuma T, Yasuda M, Fujiwara K (2014). Alpha-fetoprotein-producing ovarian clear cell adenocarcinoma simulating fetal gut in a postmenopausal woman. Gynecol Oncol Case Rep.

[4] Faure Conter C, Xia C, Gershenson D, Hurteau J, Covens A, Pashankar F, Krailo M, Billmire D, Patte C, Fresneau B, Shaikh F, Stoneham S, Nicholson J, Murray M, Frazier AL (2018). Ovarian yolk sac tumors; does age matter?. Int J Gynecol Cancer.

[5] Goyal LD, Kaur S, Kawatra K (2014). Malignant mixed germ cell tumour of ovary--an unusual combination and review of literature. J Ovarian Res.

[6] Dai CY, Lin CY, Tsai PC, Lin PY, Yeh ML, Huang CF, Chang WT, Huang JF, Yu ML, Chen YL (2018). Impact of tumor size on the prognosis of hepatocellular carcinoma in patients who underwent liver resection. J Chin Med Assoc.

[7] Konishi I, Fujii S, Kataoka N, Noda Y, Okamura H, Yamabe H (1988). Ovarian mucinous cystadenocarcinoma producing alpha-fetoprotein. Int J Gynecol Pathol.

[8] Maida Y, Kyo S, Takakura M, Kanaya T, Inoue M (1998). Ovarian endometrioid adenocarcinoma with ectopic production of alpha-fetoprotein. Gynecol Oncol.

[9] Meguro S, Yasuda M (2013). Alpha-fetoprotein-producing ovarian tumor in a postmenopausal woman with germ cell differentiation. Ann Diagn Pathol.

[10] Tsung JSH, Yang PS (2004). Hepatoid carcinoma of the ovary: characteristics of its immunoreactivity. A case report. Eur J Gynaecol Oncol.

[11] Senzaki H, Kiyozuka Y, Mizuoka H, Yamamoto D, Ueda S, Izumi H (1999). An autopsy case of hepatoid carcinoma of the ovary with PIVKA-II production: immunohistochemical study and literature review. Pathol Int.

[12] Yigit S, Uyaroglu MA, Kus Z, Ekinci N, Oztekin O (2006). Hepatoid carcinoma of the ovary: immunohistochemical finding of one case and literature review. Int J Gynecol Cancer.

[13] Lee CH, Huang KG, Ueng SH, Swei H, Chueh HY, Lai CH (2002). A hepatoid carcinoma of the ovary. Acta Obstet Gynecol Scand.

[14] Tejerina Gonzalez E, Arguelles M, Jimenez-Heffernan JA, Dhimes P, Vicandi B, Pinedo F (2008). Cytologic features of hepatoid carcinoma of the ovary: a case report with immunocytologic evaluation of HepPar1. Acta Cytol.

[15] Matsuta M, Ishikura H, Murakami K, Kagabu T, Nishiya I (1991). Hepatoid carcinoma of the ovary: a case report. Int J Gynecol Pathol.

[16] Tochigi N, Kishimoto T, Supriatna Y, Nagai Y, Nikaido T, Ishikura H (2003). Hepatoid carcinoma of the ovary: a report of three cases admixed with a common surface epithelial carcinoma. Int J Gynecol Pathol.

[17] Abe A, Furumoto H, Yoshida K, Nishimura M, Irahara M, Kudo E (2008). A case of ovarian endometrioid adenocarcinoma with a yolk sac tumor component. Int J Gynecol Cancer.

[18] Horiuchi A, Osada R, Nakayama K, Toki T, Nikaido T, Fujii S (1998). Ovarian yolk sac tumor with endometrioid carcinoma arising from endometriosis in a postmenopausal woman, with special reference to expression of alpha-fetoprotein, sex steroid receptors, and p53. Gynecol Oncol.

[19] Kamoi S, Ohaki Y, Mori O, Okada S, Seto M, Matsushita N (2002). A case of ovarian endometrioid adenocarcinoma with yolk sac tumor component in a postmenopausal woman. APMIS.

[20] Nomura K, Miyasaka Y, Murae M, Terashima Y, Aizawa S (1992). Ovarian mucinous cystadenocarcinoma producing alpha-fetoprotein. A case report. Acta Pathol Jpn.

[21] Arai T, Kitayama Y, Koda K (1999). Ovarian mucinous cystadenocarcinoma with yolk sac tumor in a 71-year-old woman. Int J Gynecol Pathol.

[22] Suzuki T, Ino K, Kikkawa F, Shibata K, Kajiyama H, Morita T (2003). Cushing’s syndrome due to ovarian serous adenocarcinoma secreting multiple endocrine substances: a case report and immunohistochemical analysis. Gynecol Oncol.

[23] Higuchi Y, Kouno T, Teshima H, Akizuki S, Kikuta M, Ohyumi M (1984). Serous papillary cystadenocarcinoma associated with alpha-fetoprotein production. Arch Pathol Lab Med.

[24] Isonishi S, Ogura A, Kiyokawa T, Suzuki M, Kunito S, Hirama M (2009). Alpha-fetoprotein (AFP)-producing ovarian tumor in an elderly woman. Int J Clin Oncol.

[25] Hu JM, Chou YC, Wu CC, Hsiao CW, Lee CC, Chen CT, Hu SI, Liu WT, Jao SW (2016). Adjuvant chemotherapy with tegafur/uracil for more than 1 year improves disease-free survival for low-risk stage II colon cancer. J Chin Med Assoc..

[26] Bayrak R, Haltas H, Yenidunya S (2012). The value of CDX2 and cytokeratins 7 and 20 expression in differentiating colorectal adenocarcinomas from extraintestinal gastrointestinal adenocarcinomas: cytokeratin 7−/20+ phenotype is more specific than CDX2 antibody. Diagn Pathol.

[27] Esheba GE, Pate LL, Longacre TA (2008). Oncofetal protein glypican-3 distinguishes yolk sac tumor from clear cell carcinoma of the ovary. Am J Surg Pathol.

[28] Kato N, Motoyama T (2009). Expression of hepatocyte nuclear factor-1beta in human urogenital tract during the embryonic stage. Anal Quant Cytol Histol.

[29] Ye S, Yang J, You Y, Cao D, Huang H, Wu M, Chen J, Lang J, Shen K (2016). Clinicopathologic significance of HNF-1β, AIRD1A, and PIK3CA expression in ovarian clear cell carcinoma: a tissue microarray study of 130 cases. Medicine (Baltimore).

[30] Huang W, Cheng X, Ji J, Zhang J, Li Q (2016). The application value of HNF-1β transcription factor in the diagnosis of ovarian clear cell carcinoma. Int J Gynecol Pathol.

[31] Suzuki E, Kajita S, Takahashi H, Matsumoto T, Tsuruta T, Saegusa M (2015). Transcriptional upregulation of HNF-1β by NF-κB in ovarian clear cell carcinoma modulates susceptibility to apoptosis through alteration in bcl-2 expression. Lab Investig.

[32] Sahin H, Meydanli MM, Sari ME, Yalcin I, Çoban G, Ozkan NT, Cuylan ZF, Erdem B, Gungorduk K, Akbayir Ö, Dede M, Salman MC, Güngör T, Ayhan A (2018). Does the primary route of spread have a prognostic significance in stage III non-serous epithelial ovarian cancer?. J Ovarian Res..

[33] Sung PL, Wen KC, Horng HC, Chang CM, Chen YJ, Lee WL, Wang PH (2018). The role of α2,3-linked sialylation on clear cell type epithelial ovarian cancer. Taiwan J Obstet Gynecol.

